# Tailoring of the Properties of Amorphous Mesoporous Titanosilicates Active in Acetone Condensation

**DOI:** 10.3390/gels10110732

**Published:** 2024-11-12

**Authors:** Vera R. Bikbaeva, Anna S. Artem’eva, Sergey V. Bubennov, Alexander I. Nikiforov, Viktor Y. Kirsanov, Dmitry V. Serebrennikov, Lubov F. Korzhova, Stanislav G. Karchevsky, Leonard M. Khalilov, Boris I. Kutepov, Nellia G. Grigoreva

**Affiliations:** 1 Institute of Petrochemistry and Catalysis of the Ufa Federal Research Center, The Russian Academy of Sciences, Ufa 450075, Russia; vrbikbaeva@mail.ru (V.R.B.); artones@mail.ru (A.S.A.); bubennov@list.ru (S.V.B.); zorge31-3@mail.ru (V.Y.K.); d25c25@yandex.ru (D.V.S.); khalilovlm@gmail.com (L.M.K.); kutepoff@inbox.ru (B.I.K.); 2 Department of Chemistry, Lomonosov Moscow State University, Moscow 119991, Russia; alexandernikiforov@mail.ru; 3 Joint Stock Company “Institute of Petrochemical Processing” (JSC “INHP”), Ufa 450065, Russia; klf-inhp@mail.ru (L.F.K.); st_karchevsky@mail.ru (S.G.K.)

**Keywords:** amorphous titanosilicate, sol–gel, template-free synthesis, TiO_2_, heterogeneous catalyst, acetone condensation, mesitylene

## Abstract

Amorphous mesoporous materials are promising as catalysts for processes involving or forming bulk molecules. In a reaction such as acetone condensation to form mesitylene, an effective catalyst should not only have a developed porous structure but also have active centers of acidic and basic types. The sol–gel approach allows one to obtain titanosilicates with such characteristics. This work demonstrates the possibility of controlling their properties by varying the conditions for the synthesis of titanosilicate gels. It has been established that controlling hydrolysis allows one to increase the activity of amorphous mesoporous titanosilicates by 10 times: from acetone conversion of 6% to 60%. It has been shown that the use of titanium acetylacetonate complexes in the synthesis of gels leads to an increase in the content of tetracoordinated Ti in the structure and contributes to an increase in the acidity of titanosilicates. During the condensation of acetone on the obtained mesoporous titanosilicates, high acetone conversion (60–79%) and mesitylene selectivity of up to 83% were achieved.

## 1. Introduction

Mesoporous silica or metal–silicalite materials are in great demand. They are used as bases for catalysts, carriers for drugs [1], adsorbents for the removal of water pollutants [2], etc.

Among titanosilicates, crystalline molecular sieves, such as TS-1, used as a catalyst for oxidation processes, are the most widely known. It is possible to use titanosilicates as adsorbents of heavy metals [3], for CO_2_ capture [4]. In contrast to SiO_2_-TiO_2_ composite materials, titanium in titanosilicates is incorporated in the structure and is not in the final material as a separate phase.

Amorphous titanosilicates and their properties are poorly studied [5,6,7]. These systems have a number of advantages over their crystalline counterparts, such as greater availability of active sites, easier separation from the reaction mass than for nanoscale crystals, and low cost. Sol–gel synthesis opens wide possibilities for the regulation of properties of noncrystalline titanosilicates.

The synthesis of metal silicates differs from the synthesis of systems based on silica alone. Regulation and control of hydrolysis rates, as well as the subsequent condensation of silica and metal sources, are necessary conditions for obtaining a homogeneous structure.

Tetraethyl orthosilicate (TEOS) is one of the most popular Si sources for metal silicates [8,9,10]. The kinetics of the hydrolysis and condensation step of TEOS directly also depends on the solvent or solvent mixture nature [11], pH value, and catalyst presence. TEOS hydrolysis can be manipulated by directly changing the pH medium or by adding a catalytic amount of H^+^ or OH^−^ sources. Acidic or basic tetraethyl orthosilicate hydrolysis can be performed via various routes. As noted from the literature, acid-catalyzed hydrolysis of the TEOS results in the weakly cross-linked gel, base-catalyzed hydrolysis results in a high number of branched clusters [12]. In other words, under pH < 7, the hydrolysis step occurs at a high rate; however, the following condensation would be slow. Under basic conditions, both steps are fast.

The conditions under which the silicon source is introduced can affect the rate and direction of hydrolysis of the titanium source. One of the problems in the synthesis of titanosilicates is to prevent the formation of SiO_2_ and TiO_2_ phases separately. As a titanium source, it is convenient to use its alkoxides. However, for example, titanium isopropoxide (TTIP) is also widely used in the synthesis of titanium dioxide [13,14]. The rate of hydrolysis and precipitation of Ti(OH)_4_, and subsequent condensation to form TiO_2_xH_2_O in the presence of water, is affected by the molar ratio between H_2_O and Ti [15], pH [16], and the depth of hydrolysis [14]. Similar approaches are used for the controlled introduction of titanium into the silicate structure in the synthesis of titanosilicate molecular sieves. However, it is emphasized that it is necessary to create conditions that prevent the transformation of titanium alkoxides into TiO_2_.

The synthesis of amorphous titanosilicate with the principal pH modification was described in a limited number of works [6,7]. In the first article, a catalytic amount of hydrochloric acid was added for hydrolysis acceleration (TEOS:HCl molar ratio was 1:9 × 10^−4^). After, the authors stopped the hydrolysis through the influx of NH_4_OH solution. However, as the final substance was silylating, no information about the characteristics of the original xerogel is available. In the second work [7], the authors compared the physicochemical properties of the amorphous titanosilicates obtained at pH = 3 and pH = 10 with the transition pH from 3 to 10. The pH value for the hydrolysis step crucially affected the porosity and Ti coordination.

The formation of active sites in titanosilicates is usually considered from the point of view of their activity for oxidative processes. However, it is known that crystalline titanosilicate ETS-10 shows basic properties [17]. Aldol condensation of acetone on basic ETS-10 leads to the formation of α,β-mesityloxides.

We have previously shown that amorphous mesoporous titanosilicate is active in the condensation of acetone and allows the production of mesitylene with 52–70% selectivity (acetone conversion 13–52%) [18]. In this connection, it was of interest to investigate the factors influencing the porous characteristics of titanosilicates, phase composition, titanium introduction, and formation of active sites of different types in them.

In the present paper, the results obtained during the synthesis of amorphous titanosilicates using different methods of synthesis, molar ratio Si/Ti, type, and concentration of active sites are considered. Physicochemical characteristics and catalytic properties of the obtained titanosilicates in aldol condensation of acetone have been studied in detail.

## 2. Results and Discussion

### 2.1. Physicochemical Properties of Catalysts

In this study, we aimed to determine the key parameters for controlling the synthesis of gels that are subsequently transformed into catalytically active amorphous mesoporous titanosilicates (TSMs). The synthesis procedure is described in detail in Section 4.

Tailoring of the Si source hydrolysis was investigated first. Aqueous solutions of HNO_3_ (for TSMa samples) and NH_4_OH (for TSMb samples) were used as hydrolysis catalysts. It is considered that the hydrolysis of tetraethyl orthosilicate is more complete in an acidic medium than in a basic medium. In the context of titanosilicate synthesis, this affects the hydrolysis rate of the titanium source. Improving the incorporation of titanium atoms into the silicate network is possible by equalizing the hydrolysis rates of the silicon source and titanium at the gel formation stage.

**Figure 1:**
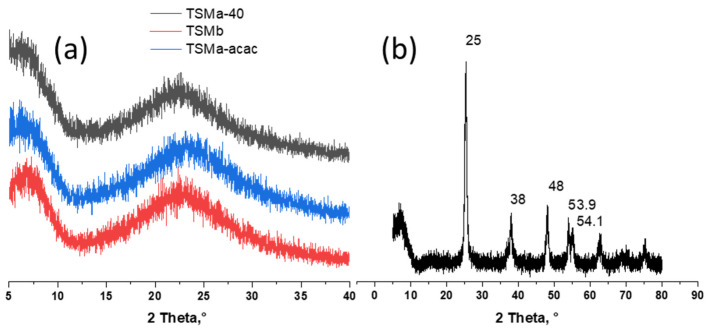
X-ray diffraction (XRD) patterns of (**a**) amorphous mesoporous titanosilicates prepared with HNO_3_ (TSMa-40 and TSMa-acac), with NH_4_OH (TSMb) during the TEOS hydrolysis step, (**b**) crystalline TiO_2_. For all titanosilicates, the Si/Ti = 40.

When titanium isoporoxyde alcohol solution was added to the silica sol prepared with an ammonia solution (Si/NH_4_OH = 200/1), a low amount of white precipitation appeared. It means that the rate of hydrolysis of titanium alkoxide was very high. As a result, part of the titanium turned into Ti(OH)_4_ [19]. When it was added to the sol prepared with nitric acid (Si/HNO_3_ = 200/1), the resulting new sol was fully transparent and homogeneous. Further incorporation of titanium into the silicate network for the TSMb sample was difficult. This was further confirmed by UV-Vis and UV–Raman techniques (Figure 1).

It should be noted that the Si/Ti ratio affects the gelation time. With a decrease in Ti content, the time required before the onset of gelation of sols increases. For example, for samples with Si/Ti = 20 (TSMa-20), this parameter is equal to 15 h; for the sample with Si/Ti = 80 (TSMa-80), it is close to 30 h.

For the sample TSMa-acac, the rate of hydrolysis of titanium isopropoxide was additionally slowed down by forming a complex with acetyl acetone [20].

In addition to titanosilicates, TiO_2_ was obtained as a comparison sample in this study.

The physicochemical characteristics of the obtained samples are discussed below.

#### 2.1.1. Phase Composition Study

The transformation of titanosilicate sols into gel took place at low temperatures (60 °C), at which the formation of crystalline products was not observed. The subsequent treatment stage of the obtained gel with ammonia solution also does not lead to the formation of new phases. At final calcination, amorphous titanosilicate does not crystallize. This fact indirectly proves the absence of other Ti-containing compounds. All TSM samples were X-ray amorphous (Figure 1a).

In contrast to titanosilicates, the final formation of titanium dioxide from precipitated amorphous Ti(OH)_4_ occurs during calcinations [21]. The results for the TiO_2_ sample are shown in the figure (Figure 1b). The X-ray diffraction patterns showed the presence of peaks at 25, 38.48, 53.9, and 54.1°. All these peaks were attributed to anatase. No traces of the rutile phase were found.

#### 2.1.2. Morphology Study

**Figure 2:**
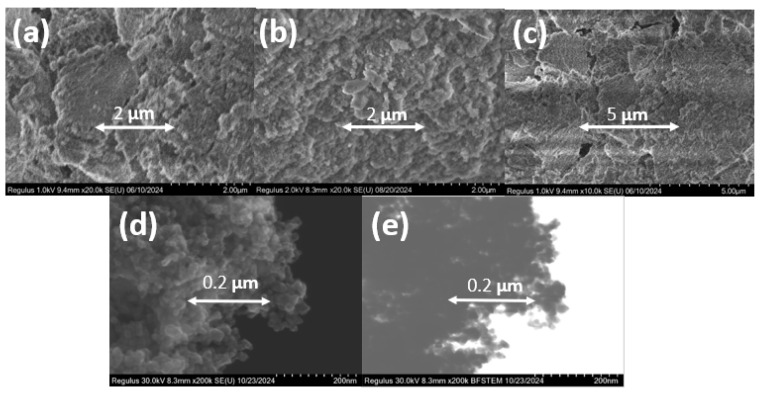
SEM images of various types of the amorphous and crystalline titanosilicates synthesized with a similar Si/Ti ratio: (**a**) TSMa-40, Si/Ti = 40; (**b**) TSMa-acac, Si/Ti = 40; (**c**) TSMb, Si/Ti = 40; (**d**) TiO_2_; (**e**) STEM image of TiO_2_.

Different titanosilicates of each type (TSMa-40, TSMa-acac, TSMb, Si/Ti = 40) and titanium dioxide were examined by scanning electron microscopy (Figure 2). All amorphous samples are represented by «fluffy» bulk aggregates with extremely homogeneous structures and without rigid aggregations. The crystallite size of 20–40 nm for TiO_2_ was determined from SEM and STEM images. All spherical particles were aggregated with each other.

#### 2.1.3. Porosity Study

The effect of the presence of H^+^ or OH^−^ catalyst during the hydrolysis step of TEOS on the porous structure was investigated by low-temperature N_2_ adsorption–desorption (Figure S1). According to the methodology used, no OSDA solid templates are applied. The formation of mesoporosity occurs during the degradation of the titanosilicate gel by an abrupt change in pH.

Both samples, TSMa-40 and TMSb, were mesoporous and were obtained with IV-type isotherms. However, a fundamental difference in the total pore volume (V_total_) is present. For the TSMa-40 sample, V_total_ = 0.97 cm^3^/g, while for TSMb, it is about 10 times smaller (0.09 cm^3^/g). The specific surface area for TSMa is 600 m^2^/g and for TMSb, about 36 m^2^/g.

#### 2.1.4. Titanium State and Active Sites Formation Study

Titanium states in the samples were determined by UV-Vis Diffuse Reflectance Spectroscopy. The formation of structured and unstructured Ti-species was determined by UV–Raman Spectroscopy.

**Figure 3:**
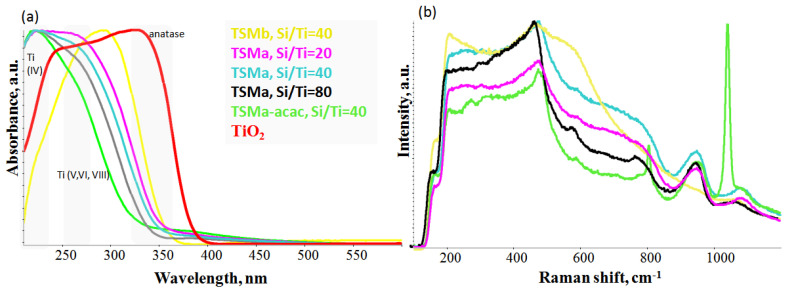
UV-vis absorption spectra (**a**) and UV–Raman spectra (**b**) of amorphous mesoporous titanosilicates TSMb, TSMa-20, TSMa-40, TSMa-80, TSMa-acac, and TiO_2_.

The UV-vis spectra of titanosilicate samples (Figure 3a) usually show several absorption bands. Closed Ti(OSi)_4_ sites are characterized by a band at 200–220 nm. Open Ti-species in a tetrahedral environment have been correlated with a band at 228–235 nm [22]. Pentahedral and octahedral coordinated Ti with bands in the range of 250–280 nm can also be present in titanosilicates [23]. On the other side, the authors of [24] consider that the band at 235 nm refers to pentacoordinated Ti-species, and in [25], the band at 260–280 nm refers to absolutely symmetric vibrations of hexacoordinated Ti-O-Ti bonds of amorphous Ti particles. For all papers devoted to the study of titanosilicates, a common trend is observed: an increase in the Ti content in the sample leads to an increase in the band at 310–330 nm, which is the characteristic band of TiO_2_ anatase.

In the series of TSMa samples (Si/Ti = 20, 40, 80), signals from the Ti are observed in different environments. There is no anatase in the whole series of samples, as indicated by the absence of an absorption band at 330 nm. The bands shifting to a longer wavelength region for the series indicates that there is an increase in the amount of titanium in V and/or VI coordination. It is assumed that the number of anchored titanium to surface or surface hexacoordinated Ti(OH)_2_(H_2_O)_2_(OSi)_2_ sites is likely to increase.

The TSMa-acac sample has the largest amount of tetracoordinated titanium Ti(IV) compared to the TSMa series (Si/Ti = 20–80). At the same time, based on the UV–Raman spectra (Figure 3b), new active sites are present. Based on the literature data, it can be assumed that this sample has open Ti(OH)(OSi)_3_ and closed Ti(OSi)_4_ active sites [26]. UV-Vis bands for these sites are 220–235 nm and 200 nm, respectively.

There is no tetracoordinated titanium Ti(IV) in the spectrum of the TSMb sample. Probably, it represents amorphous TiO_2_ or fixed titanium on the surface of silicalite.

It is known that framework titanium in the crystalline titanosilicates has characteristic bands at 960 and 1125 cm^−1^ in the Raman spectra. These bands are absent in pure silicate-1 and can be used for qualitative determination of Ti coordination and incorporation degree. For example, TS-1 spectra usually also have structural silicate-1 bands: 290, 375, 467, 799–828 cm^−1^, and when increasing the Ti content appear strips at 140–144, 386–400, 513–516, and 637–639 cm^−1^, which are characteristic for extra-framework titanium as TiO_2_ [27,28].

The Raman spectra of the amorphous TSMa series (Si/Ti = 20–80) differ slightly (Figure 3b). On the one hand, they do not show signals from the MFI structure (as TS-1); on the other hand, there are no signals from anatase TiO_2_ (393, 514, and 635 cm^−1^). The broad signal at 940 and 1085 cm^−1^ is most likely related to the oligomeric linkages of Si-O-Si and has no relation to the fragments of the titanium.

In the TSMa-acac sample, an atypical intense signal at 1040 cm^−1^ is observed. In agreement with the UV-vis data and based on the papers [29,30], we can assume that the 1040 cm^−1^ band in the UV–Raman spectrum in amorphous materials corresponds to vibrations from the open site Ti(OH)(OSi)_3_.

#### 2.1.5. Acidity and Basicity Study

Figure S2 and Table 1 present the FTIR spectra of adsorbed pyridine for the discussed samples. All titanosilicate samples exhibit two absorption bands in the range of 1443–1510 cm^−1^, and TiO_2_ has a third band at 1544 cm^−1^. The bands at 1443–1447 cm^−1^ are attributed to pyridine adsorbed on Lewis acid sites (LAS), while the band at 1544 cm^−1^ is attributed to protonated pyridine (Brønsted acid sites, BAS). The peaks at 1490–1510 cm^−1^ contain contributions from both BAS and LAS [31].

The prepared titanosilicates contain only LAS with a concentration of 8–55 μmol pyridine∙g^−1^. Titanium dioxide (TiO_2_) demonstrated a higher concentration of Lewis acid sites compared to the titanosilicates (77 μmol pyridine∙g^−1^) and also the presence of Brønsted acid sites (4 μmol pyridine∙g^−1^). The sample with Si/Ti = 40, prepared with acid as hydrolysis catalysts (TSMa-40), has a LAS concentration 3.6 times greater than the sample synthesized with base (TSMb). The lower acidity of TSMb may be attributed to its less developed mesoporous structure (the total pore volume differs by approximately 10 times; see Figure S1) and smaller specific surface area. TSMa-acac sample demonstrates the highest concentration of acid sites, probably due to open Ti(OH)(OSi)_3_ sites.

As the Si/Ti ratio increases from 20 to 80 in the TSMa samples prepared with HNO_3_, the concentration of LAS in the titanosilicates predictably decreases (from 47 to 18 μmol pyridine∙g^−1^).

The presence of the basic sites was demonstrated by using FTIR spectroscopy with a chloroform probe molecule (Figure S3). The band about 2970 cm^−1^ relates to the interaction of the hydrogen atom of chloroform with the oxygen atoms of zeolite and metal oxides [32]. Based on the integral intensity of the specified band, one can judge which sample contains a greater number of basic sites. The basicity grew with the increasing Si/Ti ratio. Since the strength of the main basic sites is evaluated by the shift of the bands in the region of 3030–2950 cm^−1^ relative to the groups at 3033 cm^−1^ (physically adsorbed (condensed) chloroform [33]), it can be concluded that all basic sites were weak.

### 2.2. Result of Catalytic Performance

The catalytic properties of the synthesized titanosilicates were studied in the aldol condensation of acetone. TiO_2_ was used as a catalyst of comparison.

Acetone transformations in the presence of Ti-containing catalysts produced a reaction mixture containing unsaturated ketones—mesityl oxide (**MO**) and mesityl iso-oxide (**iMO**); mesitylene (**M**) and minor amounts of other trimethylbenzenes (pseudocumene **PC**, hemellitol **H**); isophorone (**iP**); acetic acid (**AA**); isobutene (**IB**); 3,5-xylenol (**3,5-DMP**); 2,3,5-trimethylphenol (**2,3,5-TMP**); tetralone (**TMT**); tetramethylcyclohexenes ΣC_10_H_16_, dialkenylbenzenes ΣC_15_H_20_, and a number of other compounds hereinafter designated as “other”: ΣC_12_H_16_ (alkenylbenzenes); ΣC_7_H_12_ (dimethylcyclopentadienes), ΣC_14_H_16_ (tetramethylnaphthalenes), 4-methylpentan-2-one; dehydroisophorone. Methane and carbon dioxide were identified in the gaseous reaction products.

**Figure 4:**
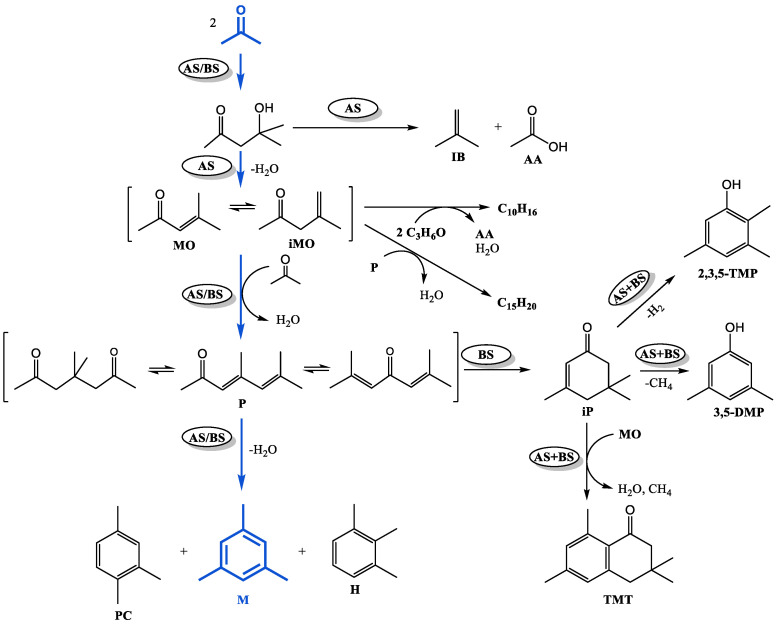
Scheme of chemical transformations of acetone on Ti-containing catalysts (AS—stage catalyzed by acidic sites; BS—stage catalyzed by basic sites).

Based on the identified composition of the reaction mass and taking into account the known literature data on the mechanism of acetone condensation [18,34], the general scheme of its chemical transformations can be presented as follows (Figure 4).

According to the generally accepted mechanism of aldol condensation of acetone, the primary product of the reaction, which can proceed with the participation of acidic or basic sites [35], is diacetone alcohol, which is further transformed into isobutene and acetic acid [36] on sufficiently strong acid sites [37,38]. Dehydration of diacetone alcohol (on acidic or basic sites) gives a mixture of α- and β-mesityl oxides, which can attach the next acetone molecule and, after dehydration, transform into phorons (acidic or basic sites are required for the reaction to proceed) [39]. Cyclocondensation of **P** on acidic sites leads to the formation of trimethylbenzenes: mesitylene, hemellitol, and pseudocumene. Cyclocondensation of phorones involving basic sites results in the formation of isophorone [34,40]. As shown in our previous work [41], in the presence of a catalyst combining acidic and basic sites, isophorone gives **3,5-DMP**, **2,3,5-TMP**, and **TMT**. When **MO** interacts with phorones or acetone (with acetic acid stripping [36]), the by-products tetramethylcyclohexenes ΣC_10_H_16_ and dialkenylbenzenes ΣC_15_H_20_ are formed. Consequently, the composition of acetone condensation products will depend on the acid–base properties of the catalyst.

The results of acetone condensation over Ti-containing catalysts are given in Table 2.

A preliminary study of the influence of Si/Ti molar ratio on the catalytic properties of titanosilicates TSMa (three samples with different ratios of Si/Ti = 20; 40; 80) in acetone condensation showed that the maximum values of conversion (60%) and selectivity (77%) were achieved on the sample TSMa-40. The results are summarized in the SM (Figure S4). Therefore, all further studies were carried out using the TSMs with a molar ratio of Si/Ti = 40. 

The most active catalyst (based on acetone conversion values) is TiO_2_, and the least active is amorphous titanosilicate TSMb. On other amorphous titanosilicates, acetone conversion reaches 44% (TSMa-acac) and 60% (TSMa-40). The high activity of TiO_2_ is apparently due to the highest concentration of acidic sites among the studied samples.

The mentioned factor, obviously, has a decisive influence on the composition of aldol condensation products. In the reaction products obtained on TiO_2_, a significant amount of mesitylene (35%), as well as ΣC_10_H_16_ (21%) and ΣC_15_H_20_ (20%), formed as a result of side reactions involving mesityloxides **MO + iMO**, were found (Figure 4). All the above compounds are formed predominantly on acidic sites. The absence of acetic acid in the products can be explained by the fact that under the conditions of the experiment, it is consumed for side oligomerization, resulting in the formation of coke [34], as well as decomposes into methane and CO_2_, which were identified in the gaseous products.

On mesoporous titanosilicates, the composition of the products is close and differs markedly from that obtained on TiO_2_. The high selectivity of mesitylene formation, amounting to 76–80% under the studied conditions, attracts attention. **TMT** (0–2%) and compounds ΣC_10_H_16_ (1–2%) and ΣC_15_H_20_ (0–1%) are present in very minor amounts, and **AA** and **IB** are absent. This indicates that the less acidic titanosilicates, possessing only LAS (Table 1), suppress the side reactions of decomposition and condensation. In addition, **MO + iMO** (especially on TSMb) and **3,5-DMP**, **2,3,5-TMP** obtained from the isophorone are formed in increased amounts compared to TiO_2_, indicating the greater basicity of titanosilicates relative to the oxide.

The decrease in acidity has a positive effect on the number of by-products, which are labeled as “others”. The amount of these compounds in TSM samples is minimal.

A comparison of the catalytic properties of titanosilicates with their structural characteristics and phase composition (Figure 3) shows that the activity of the catalysts is largely due to the presence in their structure of the tetracoordinated Ti (IV)-species. Accordingly, the TSMb sample with probably surface hexacoordinated Ti (VI)-species is less active in the reaction. Titanosilicate TSMa-acac, characterized by the presence in the structure of only Ti (IV), as well as the presence on the surface of active fragments with open, active Ti(IV)-species, is characterized by the maximum selectivity of mesitylene formation.

Thus, the optimal combination of acidic and basic sites, as well as the presence of Ti(OSi)_4_ sites in the structure of titanosilicates, allows the selective production of mesitylene on the prepared catalysts.

#### 2.2.1. Stability of Ti-Containing Catalysts

Deactivation of heterogeneous catalysts during operation is caused by the formation of compaction products that create diffusion difficulties for the movement of reactants to the active sites of the catalyst and reaction products from the catalyst into the reaction mass volume. As a result of aldol condensation of acetone, a number of compounds are formed (oligomers of isobutylene, oligomers of unsaturated ketones, alkyl- and alkenylbenzenes), which can turn into polycyclic arenes and serve as precursors of coke deposits [42].

**Figure 5:**
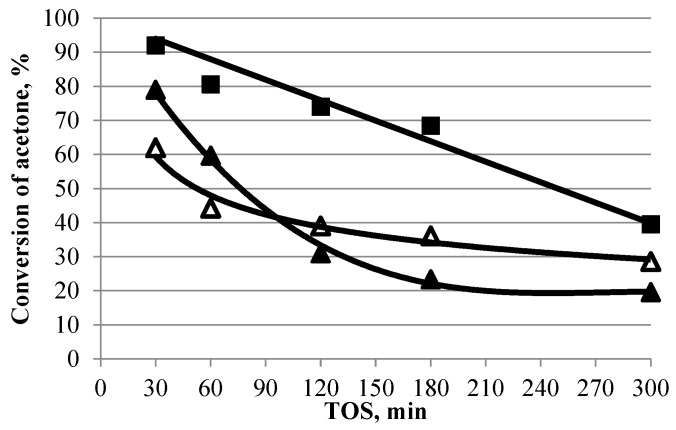
Acetone conversion over time in the presence of TiO_2_ and titanosilicates (350 °C, 1 h^−1^, TOS = 1 h). Symbol code: ■—TiO_2_, △—TSMa-acac, ▲—TSMa-40.

**Figure 6:**
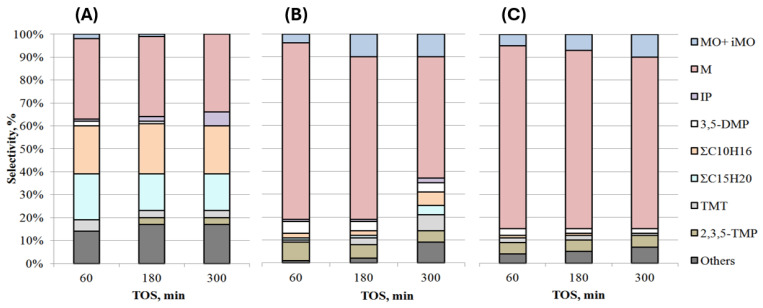
Selectivity of products over time in the presence of TiO_2_ and titanosilicates: (**A**) TiO_2_, (**B**) TSMa-40, (**C**) TSMa-acac (350 °C, 1 h^−1^, TOS = 1 h).

Changes in the activity and selectivity of Ti-containing catalysts over time in the aldol condensation of acetone are shown in Figure 5 and Figure 6. Due to the low activity of the TSMb sample, its stability was not investigated.

On samples TiO_2_ and TSMa-40, characterized by high initial activity (acetone conversion of more than 79–92%), there is a sharp decrease in acetone conversion. After 300 min, the conversion of both samples decreased by 50–60%. The most stable titanosilicate TSMa-acac, on which the conversion decreases after 300 min, is 34%.

The selectivity for mesitylene on the TiO_2_ sample practically does not change (Figure 6), there is a decrease in the amount of **MO + iMO**, **TMT**, and ΣC_15_H_20_ in the products, the content of isophorone increases, and **2,3,5-TMP** appears.

On the TSMa-40 and TSMa-acac, the selectivity for mesitylene decreases with increasing TOS, with a 24% drop in selectivity on the TSMa-40 sample and only a 5% decrease on the TSMa-acac sample. Note that on both samples, after 300 min, the **MO + iMO** content increases (from 4–5 to 10%), and in the case of TSMa-40, the content of ΣC_10_H_16_, ΣC_15_H_20_, and **TMT** increases.

The obtained results indicate that the deactivation pathways of TiO_2_ and titanosilicate samples are different. On TiO_2,_ during the reaction, deactivation of acidic sites occurs, which are responsible for the reactions accompanied by dehydration: acetone dimerization with formation of mesityl oxides **MO + iMO**, production of phorones, cyclocondensation of phorones into trimethylbenzenes, formation of ΣC_10_H_16_ and ΣC_15_H_20_, and synthesis of **TMT**. As a result, the amount of the above compounds decreases. At the same time, the share of basic-catalyzed processes increases—selectivity for isophorone increases, and **2,3,5-TMP** is formed.

On titanosilicates, the basic sites are deactivated to a greater extent; in this case, the role of acid sites is strengthened, which leads to the observed increase in the content of compounds such as **MO + iMO**, **TMT**, and by-products.

**Figure 7:**
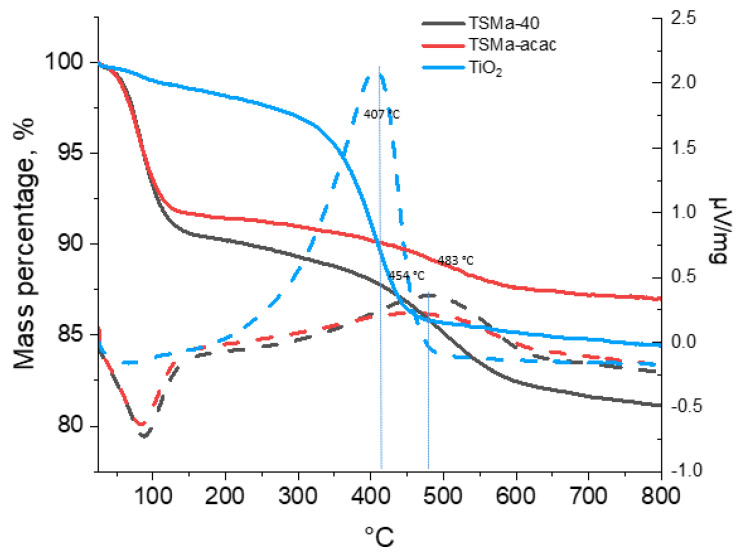
The TGA-DSC data of Ti-containing samples. Line codes: solid lines—TGA, dotted lines—DSC.

The TGA-DSC data (Figure 7) show that the greatest amount of compaction products is contained in the TiO_2_ sample (13.3%); in the samples of titanosilicates, the amount of compaction products is much less: 9.1% (TSMa-40) and 4.6% (TSMa-acacac). The most “heavy” deposits, judging by the peak with a maximum in the temperature region of 482 °C, are formed on the sample TSMa-40. On the sample TSMa-acac, lighter compaction products are formed, which follows from the lower temperatures on the derivatograph of 454 °C. The lightest coke (407 °C) is formed on the TiO_2_ sample. It is noted in [34,43] that the deactivation of TiO_2_ is caused by the adsorption of dimers and trimers obtained from acetone.

#### 2.2.2. Influence of Reaction Conditions

**Figure 8:**
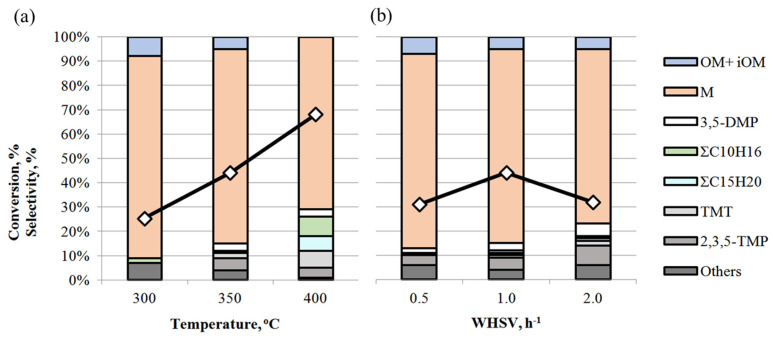
Effect of reaction conditions on acetone conversion (◇) and product selectivity over TSMa-acac: (**a**) temperature (conditions: 1 h^−1^, TOS = 1 h); (**b**) WHSV (conditions: 350 °C, TOS = 1 h).

The influence of reaction conditions (temperature and WHSV) was studied over the TSMa-acac sample, which showed the highest stability and selectivity for mesitylene **M** in acetone condensation (Figure 8).

It is shown that with increasing temperature, the conversion of acetone increases from 25% to 68% (Figure 8a). The selectivity of the reaction is maximum at 300 °C and decreases to 71% as the temperature increases to 400 °C. The decrease in selectivity is due to an increase in the content of **3,5-DMP** (from 1 to 3%), **TMT** (from 1 to 7%), ΣC_10_H_16_ and ΣC_15_H_20_, and other side compounds in the reaction mass. As we have shown earlier [18], increasing the reaction temperature promotes isomerization processes, both by double C=C bonding and by intramolecular rearrangement of methyl fragments in the benzene ring. Thus, at 400 °C, mesitylene isomers—1,2,4- and 1,2,3-trimethylbenzenes are formed, and the number of isomers of other hydrocarbons increases, and hydrogenation products appear.

Acetone conversion reaches a maximum (44%) at a WHSV of 1 h^−1^ at 350 °C (Figure 8b). The selectivity for mesitylene is 80% at WHSV = 0.5–1.0 h^−1^ and decreases to 72% at 2 h^−1^, while 3,5-DMP, 2,3,5-TMP, and TMT appear. Low conversion at WHSV 0.5 h^−1^ may indicate intensive coke formation processes on the catalyst due to longer contact time.

## 3. Conclusions

A sol–gel method for obtaining amorphous mesoporous titanosilicates characterized by complete titanium incorporation into the structure and the presence of acidic and basic sites is proposed. Due to the combination of these properties, the obtained materials demonstrated high activity and selectivity for mesitylene in the aldol condensation reaction of acetone. It was found that by controlling the sol and following gel formation, it is possible to create a mesoporous structure without introducing a template, regulate the number of acidic and basic sites, and incorporate titanium into the material structure during gel formation. The control of these processes was carried out by varying the ratio of the hydrolysis/solvolysis rates of the silicon source and titanium. It was shown that with the addition of a base, the hydrolysis rate of titanium isopropoxide is the highest. In the TSMb sample obtained by this method, no metal incorporation into silicalite in the form of tetracoordinated titanium Ti(OSi)_4_ and no anatase were detected. In the case of synthesis with the addition of acid, respectively, with a reduced difference in the rates of hydrolysis of TEOS and titanium isopropoxyde, titanium is incorporated into the metal silicate mainly as the several types of tetracoordinated species (TSMa). All TSMa samples contained closed and open tetra-coordinated Ti sites. Under the conditions of the slowest titanium source hydrolysis (TSMa-acac), when titanium was introduced via the bulky complex, the highest content of tetracoordinated titanium in the structure was observed. Moreover, the synthesis of the gel in the presence of a catalytic amount of acid allows us to obtain a mesoporous material with S_BET_ = 600 m^2^/g. Since we are talking about amorphous samples, the number of surface acidic and basic sites increases with an increase in the specific surface area. All these factors determine the advantages of titanosilicates of the TSMa type over the TSMb type.

When studying the effect of the method of synthesis of titanosilicates on their catalytic properties in the aldol condensation of acetone, it was found that the TSMa and TSMa-acac samples are most active in the reaction.

It is shown that the important factors influencing the catalytic properties of titanosilicates in the synthesis of mesitylene are the concentration of acidic and basic sites, as well as the coordination of titanium in the obtained samples. High activity, selectivity, and stability of the most effective titanosilicate TSMa-acac are due to the optimal combination of acidic and basic sites in it, as well as the presence of tetracoordinated Ti (IV) completely incorporated into the structure. In the presence of titanosilicate TSMa-acac, the conversion of acetone is 44%, and the selectivity for mesitylene is 80% (350 °C, 1 h^−1^, TOS = 1 h).

## 4. Materials and Methods

### 4.1. Synthesis of Titanosilicates via the Gels

#### 4.1.1. Mesoporous Amorphous Titanosilicate TSMa (Acid-Catalyzed)

An aqueous alcoholic solution of 10 g TEOS was pre-hydrolyzed with the addition of a catalytic amount of acid. To the resulting solution, 0.17–0.68 g of Ti(OC_2_H_5_)_4_ dissolved in isopropanol (0.17 g for the sample with Si/Ti = 80, 0.64 g for Si/Ti = 20) was slowly added at 20–25 °C. The resulting sol was sustained at 58–62 °C until gel formation. The formed gel was treated with ammonia solution. The obtained sample was heat dried in air first at 60 and then calcined at 550 °C.

#### 4.1.2. Mesoporous Amorphous Titanosilicate TSMa-Acacac (Acid-Catalyzed, Synthesized with Acetylacetone) via the Gels

An aqueous alcoholic solution of 10 g TEOS was pre-hydrolyzed with the addition of a catalytic amount of acid. To the resulting solution, 0.34 g of Ti(OC_2_H_5_)_4_ dissolved in a mixture of isopropanol and acetylacetone was slowly added at 20–25 °C. The molar ratio of acetylacetone/Ti was 5. The resulting sol was sustained at 58–62 °C until gel formation. The gel formed was treated with ammonia solution. The obtained sample was heat dried in air first at 60 and then calcined at 550 °C.

#### 4.1.3. Mesoporous Amorphous Amorphous Titanosilicate TSMb (Base-Catalyzed) via the Gels

An aqueous alcoholic solution of 10 g TEOS was pre-hydrolyzed with the addition of a catalytic amount of base (ammonia solution). An alcoholic solution of Ti(OC_2_H_5_)_4_ (0.34 g Ti(OC_2_H_5_)_4_ for the sample with Si/Ti = 40) was slowly added to the resulting solution at 20–25 °C. The resulting sol was sustained at 58–62 °C until gel formation. The formed gel was treated with ammonia solution. The obtained sample was heat dried in air first at 60 and then calcined at 550 °C.

### 4.2. Catalyst Characterization

Morphological characterization was performed using a Hitachi Regulus SU8220 scanning electron microscope (SEM) (Hitachi High-Technologies, Tokyo, Japan). The instrument is equipped with an SE detector (using a photomultiplier) and a detector for transmission (BF-STEM) with an additional diaphragm. The samples were applied to a copper grid fixed on an aluminum sample holder for SEM. Electron micrographs of all four samples were recorded at 30 kV at a magnification of 70,000×. In addition, the samples were analyzed at magnifications of 30,000×, 90,000×, and 150,000×.

The evaluation of the phase composition and degree of crystallinity of the samples were determined by X-ray diffraction (XRD) analysis with a Shimadzu XRD-7000 diffractometer (Shimadzu Corp., Tokyo, Japan) using monochromated Cu*K*_α_ radiation at angles 2θ. The measured range was from 5° to 80° with an increment of 0.5 deg/min. The diffraction patterns for TiO_2_ were interpreted by comparison with the data from the PDF2 database.

The specific surface area and the volume of micro and mesopores were determined by low-temperature (−196 °C) adsorption–desorption of nitrogen with a NOVA 1200 e (Quantachrome) sorption meter (Quantachrome, Boynton Beach, FL, USA). Before the measurements, titanosilicate samples were heat-treated under a vacuum (350 °C, 6 h). The specific surface area was calculated by the BET method at the relative partial pressure *P/P*_0_ = 0.2.

The presence of structured and extra-structured centers was determined by Raman spectroscopy. Presented spectra were recorded using a high-resolution Horiba-Jobin Yvon LabRam HR Evolution high-resolution Raman spectrometer (Horiba, Longjumeau, France) using a 355 nm laser (Cobolt Zouk 20). The laser was focused on the catalysts using a confocal microscope with an LMU-40×-UVB objective. Before experiments, laser calibration was performed using a silicon reference. For each experiment, the titanosilicate sample was ground. The Raman spectra were recorded under ambient conditions at room temperature. The figure shows normalized spectra.

The states of titanium in the samples were determined by UV-vis diffuse reflectance spectra recorded on a Shimadzu UV-3600 plus spectrometer (Shimadzu Corp., Tokyo, Japan) equipped with deuterium, tungsten, and IR lamps. Spectralon was used as a standard Labsphere. The spectrum was recorded in the range of 210–600 nm. The figure shows normalized spectra.

The acidic properties of titanosilicates were examined by FTIR spectroscopy with pyridine probe molecule (Py-FTIR). All Py-FTIR spectra were recorded with a Bruker Vertex-70 V Fourier IR spectrometer (Bruker, Karlsruhe, Germany). The spectra recording mode was 4 cm^−1^ resolution in the range 400–4000 cm^−1^. The weight-to-diameter ratio of the pellet for recording the IR spectra was 10 mg/cm^2^. The samples were calcined at 450 °C in a vacuum (0.5 Pa) for 2 h. The pyridine adsorption was performed at 150 °C and a pressure of 300 Pa for 30 min, after which the sample was evacuated at 150 °C for 30 min. The IR spectra were recorded at room temperature before adsorption and immediately after the pyridine desorption at 150 °C. The amount of BAS was quantitatively evaluated by integration of the peak at 1515–1565 cm^−1^, and the amount of LAS by integration of the peak at 1435–1470 cm^−1^, proceeding from the known integral molar extinction coefficients of pyridine for sites of each type [31].

The basic properties were evaluated by IR spectroscopy of adsorbed chloroform acting as a probe. The chloroform adsorption was performed at 25 °C and a pressure of 400 Pa for 1 h, which was followed by evacuation for 1 h at 25 °C.

### 4.3. Catalytic Performance

Acetone condensation (99%, Acros Organics, Geel, Belgium) was carried out in a plug flow reactor in the presence of TiO_2_ and titanosilicates (1 g) at 300–400 °C with a WHSV of 0.5–2 h^−1^ for 30–300 min. Before the experiment, the catalyst was calcined for 1 h in a nitrogen stream at the reaction temperature. Liquid products of catalytic conversion of acetone were analyzed with gas–liquid chromatography on a Chromatec Crystal 5000 chromatograph (Chromatec, Russia, Yoshkar-Ola) (capillary column 50 m × 0.20 mm, phase HP-1, programmed temperature 50–290 °C, carrier gas—helium, FID detector). Mass spectra of the compounds were obtained using a SHIMADZU GCMS-QP2010Plus chromatograph–mass spectrometer (Shimadzu Corp., Tokyo, Japan) (SPB-5 phase, capillary column 30 m × 0.25 mm, carrier gas—helium, programming temperature 40–300 °C, ion source temperature 200 °C, ionization energy 70 eV).

The acetone conversion (X, %) and the selectivity of products (S_i_, %) were calculated as follows:

(1) $$X\left(\%\right)\;=100\%\cdot \frac{C_{ac\;in}-C_{ac\;out}}{C_{ac\;in}},$$

$C_{ac\;in}$, $C_{ac\;out}$—the concentrations of acetone before and after the reaction, respectively;

(2) $$S\left(\%\right)\;=100\%\cdot \frac{C_{i}}{\sum C_{i}},$$

$C_{i}$—the concentration of the i-th product in the reaction mixture; $\sum C_{i}$—the total concentration of all products.

## Figures and Tables

**Table 1:** BAS and LAS concentrations in Ti-containing samples based on IR spectra of adsorbed pyridine.

Sample	Acid Sites Concentration, μmol∙pyridine g^−1^		BAS/LAS
	BAS	LAS	
TiO_2_	4	77	0.05
TSMb	0	8	-
TSMa-40	0	29	-
TSMa-acac	0	55	-
TSMa-20	0	47	-
TSMa-80	0	18	-

**Table 2:** Acetone condensation over Ti-containing catalysts (350 °C, 1 h^−1^, TOS = 1 h).

Catalyst	TiO_2_	TSMb	TSMa-40	TSMa-acac
Acetone conversion, %	81	6	60	44
Selectivity, %				
MO + iMO	2	15	4	5
M	35	76	77	80
Ps + H	1	0	traces	0
IP	1	0	1	traces
IB	0	0	0	0
AA	traces	0	0	0
3,5-DMP	2	0	5	3
ΣC_10_H_16_	21	2	2	1
ΣC_15_H_20_	20	0	1	0
TMT	5	0	1	2
2,3,5-TMP	traces	traces	8	5
Others	13	7	1	4

## Data Availability

The original contributions presented in the study are included in the article/Supplementary Materials; further inquiries can be directed to the corresponding author.

## Supplementary Materials

The following supporting information can be downloaded at: https://www.mdpi.com/article/10.3390/gels10110732/s1, Figure S1: N_2_ adsorption–desorption isotherms of amorphous titanosilicates TSMa-40 and TSMb; Figure S2: FTIR spectra of adsorbed pyridine for the titanosilicates; Figure S3: FTIR spectra of adsorbed chloroform for the titanosilicates; Figure S4: Effect of Si/Ti molar ratio on acetone conversion and selectivity of products.
