# Supplementary material for: Tailoring of the Properties of Amorphous Mesoporous Titanosilicates Active in Acetone Condensation
Source: Gels. 2024 Nov 12;10(11):732. doi: 10.3390/gels10110732 (PMC11593900; doi:10.3390/gels10110732)
Supplement: Supplementary file 1 [file gels-10-00732-s001.zip › gels-3263994-supplementary.pdf]

Supporting information for

Tailoring of the properties of amorphous mesoporous titanosilicates active in acetone condensation

Vera R. Bikbaeva [1], Anna S. Artem'eva [1], Sergey V. Bubennov [1], Alexander I. Nikiforov [2], Viktor Y. Kirsanov [1,3], Dmitry V. Serebrennikov [1], Lubov F. Korzhova [3], Stanislav G. Karchevsky [3], Leonard M. Khalilov [1], Boris I. Kutepov [1], Nellia G. Grigoreva [1,*]

[1] Institute of Petrochemistry and Catalysis of the Ufa Federal Research Center of the Russian Academy of Sciences, Ufa 450075, Russia

[2] Department of Chemistry, Lomonosov Moscow State University, Moscow 119991, Russia

[3] Joint stock company "Institute of petrochemical processing" (JSC "INHP"), Ufa 450065, Russia

*Correspondence: ngg-ink@mail.ru

Figures

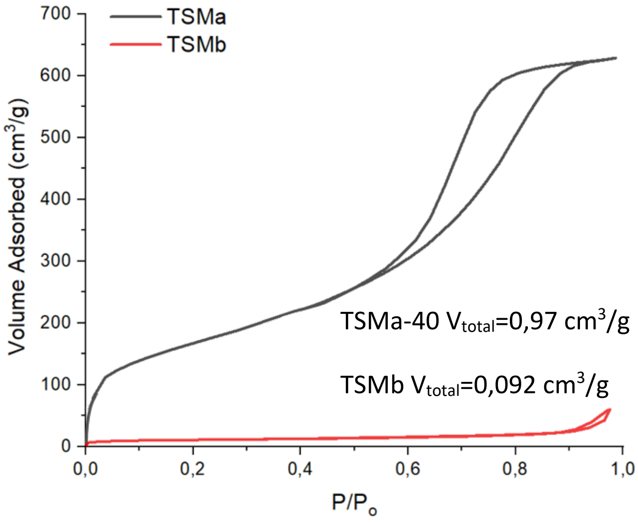

Figure S1. N_2 adsorption-desorption isotherms of amorphous titanosilicates TSMa-40 and TSMb.

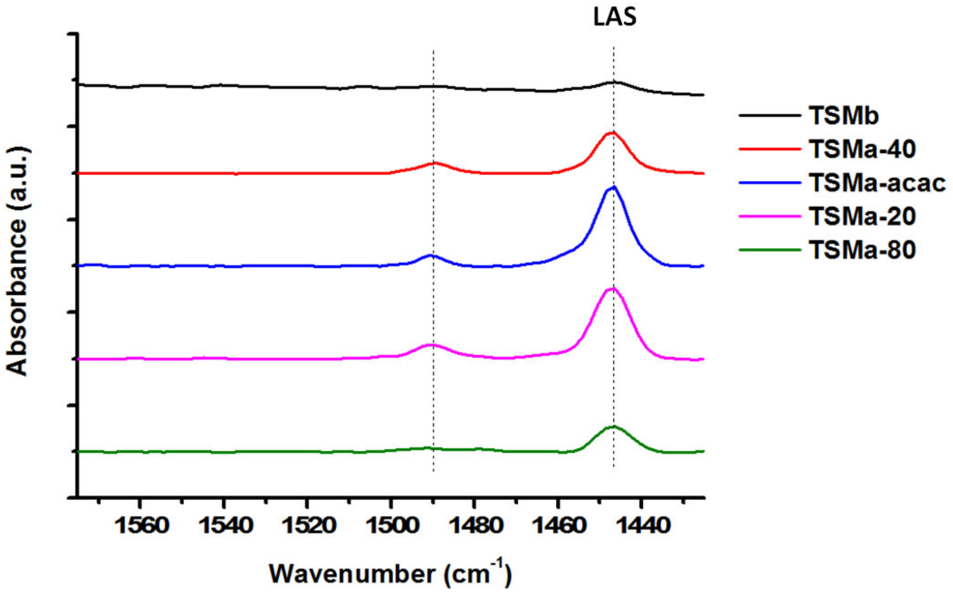

Figure S2. FTIR spectra of adsorbed pyridine for the titanosilicates

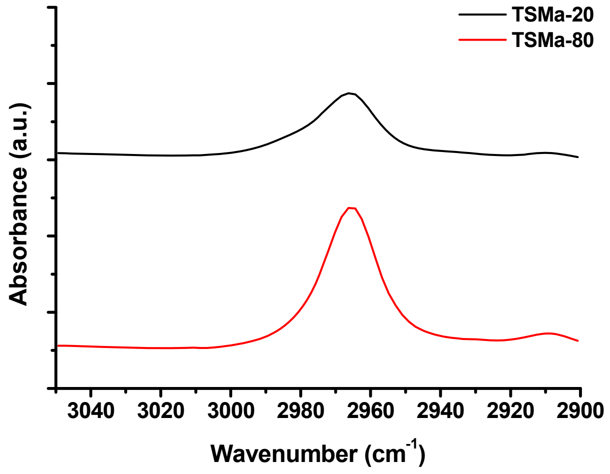

Figure S3. FTIR spectra of adsorbed chloroform for the titanosilicates

Effect of Si/Ti molar ratio

The influence of Si/Ti molar ratio on the catalytic properties of titanosilicates in acetone condensation was studied using samples with different ratios of framework atoms: Si/Ti= 20; 40; 80. The results are shown in Fig.S4.

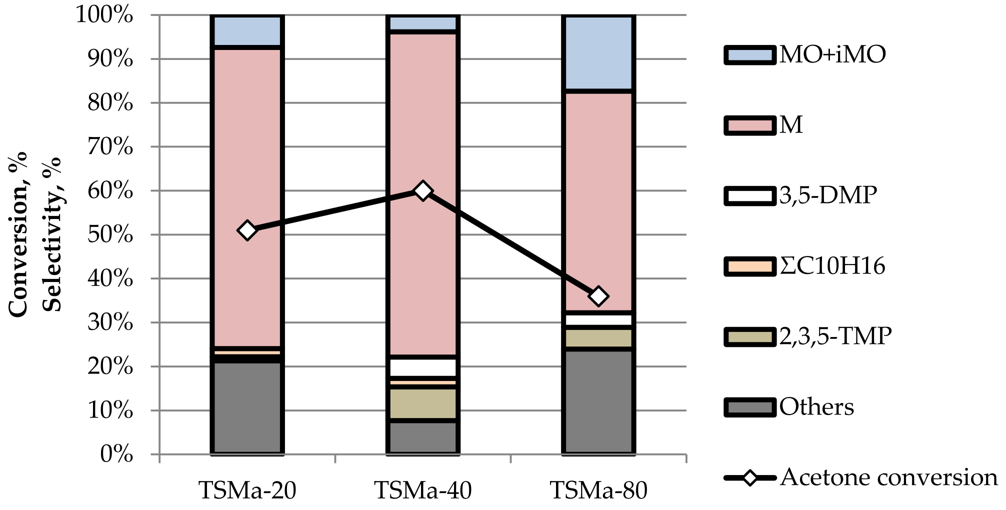

Figure S4. Effect of Si/Ti molar ratio on acetone conversion and selectivity of products (350 ^{o}C, 1 h^{-1}, TOS = 1h)

The acetone conversion curve has an extreme character, the maximum conversion were reached on sample TSMa-40 (60%), the minimum conversion on sample TSMa-80 (36%).

The most selective (77%) mesitylene is formed on sample TSMa-40, a close value of 74% was achieved on sample TSMa-20. It should be noted that the amount of mesitylene oxides and products of side reactions ($\Sigma C_{12}H_{16}$, $\Sigma C_{15}H_{20}$, $\Sigma C_{7}H_{12}$, $\Sigma C_{13}H_{20}$) increases in the reaction products as the amount of titanium in the structure of titanosilicates decreases.

The obtained results (maximum activity and selectivity in the reaction of sample TSMa-40) are obviously due to the structural characteristics of the samples. In TSMa samples with Si/Ti ratios from 20 to 80, tetracoordinated titanium Ti(IV) is predominantly present. As it was found (Figure 3), the content of hexacoordinated titanium increases with decreasing Si/Ti ratio.
